# A retrospective cephalometric study on upper airway spaces in different facial types

**DOI:** 10.1186/s40510-017-0180-2

**Published:** 2017-08-21

**Authors:** Roselaine Sprenger, Luciano Augusto Cano Martins, Júlio Cesar Bento dos Santos, Carolina Carmo de Menezes, Giovana Cherubini Venezian, Viviane Veroni Degan

**Affiliations:** 1 Araras Dental School-UNIARARAS, Araras, SP Brazil; 2 Department of Radiology, Piracicaba Dental School, University of Campinas, Piracicaba, SP Brazil; 3 Department of Orthodontics, Araras Dental School-UNIARARAS, Araras, SP Brazil

**Keywords:** Orthodontics, Cephalometry, Nasopharynx, Oropharynx, Hypopharynx, Facial types

## Abstract

**Background:**

Craniofacial growth pattern has been correlated with variations in size of the upper airway spaces. The objective of this study was to evaluate the nasopharyngeal, oropharyngeal, and hypopharyngeal airway spaces variations according to the craniofacial growth pattern, by comparing brachyfacial, mesofacial, and dolichofacial in Angle Class I individuals.

**Methods:**

To measure the spaces, 45 lateral teleradiographs were used and divided into 3 groups per the craniofacial growth pattern, determined by the Tweed cephalometry angular measurements: FMA and Y-axis. To evaluate the airways, sleep apnea cephalometry was used, containing 28 points that compose 14 factors. Three groups were compared relative to each of the 14 sleep apnea cephalometry measurements. Adherence test to the normal curve was performed. For the non-normally distributed data—measurement of the inferior pharyngeal space—the Kruskal-Wallis test was used for comparison between the groups. For the remaining data, the distribution was normal and ANOVA test was used.

**Results:**

Statistically significant difference was verified among the groups for the measurement of the median posterior-palatal space, with the difference being pointed out by the post hoc test between the brachyfacial and dolichofacial groups. For the other measurements, there was no statistically significant difference.

**Conclusions:**

It could be concluded that there was difference in the median posterior-palatal space measurement, in the oropharynx region, which was reduced for individuals with a dolichofacial pattern.

## Background

The upper airway is composed of the nasopharynx, oropharynx, and hypopharynx. Pharyngeal space size is determined primarily by the relative growth and size of the soft tissues surrounding the dentofacial skeleton [1, 2].

A normal upper airway improves nasal breathing and is considered important in the growth and development of craniofacial structures [1, 2].

An obstructive upper airway is present when obstructive processes of a morphological, physiological, or pathological nature occur, such as hypertrophy of adenoids and tonsils, chronic and allergic rhinitis, irritant environmental factors, infections, congenital nasal deformities, nasal traumas, polyps, and tumors cause functional imbalance and result in oral breathing patterns [3].

The upper airway dimensions may be influenced by the facial skeletal pattern, in which the relationship between the position of the maxilla and mandible in the anteroposterior direction has great influence on space [1].

There are studies in the literature about changes in the upper airways resulting from orthodontic treatment, orthognathic surgery or in individuals diagnosed with sleep apnea [4–7]; however, few studies have shown evidence of the airspace related to facial types and Angle Class I individuals, and this information is relevant to assist in orthodontic planning.

The aim of the present study was to evaluate the nasopharyngeal, oropharyngeal, and hypopharyngeal airway spaces in brachyfacial, mesofacial, and dolichofacial in Angle Class I individuals.

## Methods

### Sample characteristics and data collection

The sample size calculation for the difference between two measurements was made considering the test power of 80 and 95% confidence coefficient. The values with reference to the mean and standard deviation of the variable median posterior palatal space when compared with two independent groups, and the estimate of the minimum difference to be detected, required for the calculation, were retrieved from the previous results [8].

The sample was composed of 45 digital lateral head teleradiographs of adult individuals, with permanent dentition, skeletal Class I determined by Steiner’s variable ANB [8], mean SNA of 82.65° (standard deviation = 1.94), and SNB 81.11° (standard deviation 1.95).

Excluded from the study were teleradiographs lacking distinctness of structures in the image, previous history of palatine tonsil and/or pharyngeal tonsil surgeries, orthodontic treatment and/or orthognathic surgeries, volunteers submitted to extractions, or those with dental agenesis.

Teleradiographs were obtained by using the same equipment (Cranex D® Soredex Orion Corporation, Tuusula, Finland).

The cephalometric analyses were performed digitally by means of the Radiocef Studio 2 program (Radiomemory Ltda, MG, Brazil), using a resolution of 300 dpi for the teleradiographs [9]. For this purpose, the radiographs were digitized by a table scanner (Cranex D^®^ Soredex Orion Corporation, Tuusula, Finland) [10], coupled to a transparency reader, using a resolution of 300 dpi, and saved in TIFF format (*Tagged Image File Format*) without compression.

The measurements were performed by a single, blinded, duly calibrated examiner. Intra-examiner reliability was tested by re-doing 30% of the cephalometric analyses. Five teleradiographs were randomly selected from each group, totaling 15 teleradiographs of patients whose sleep apnea cephalometries were repeated after 30 days.

The Tweed FMA [11] and Y-Axis measurements were used to select the facial type. Teleradiographs were randomly selected at a radiology center and classified according to the FMA and Y-axis, and all teleradiographs presented the same FMA measures and Y-axis classification. The FMA measurement corresponded to the angle between the mandibular plane (GoMe) and the Frankfort plane (PoOr), and its reference value was 25°. Values above 30° were considered a vertical growth trend (dolichofacial); below 20°, a horizontal trend (brachyfacial), and the Y-axis, also called the angle of facial growth, formed by the sella-gnathion line and the Frankfort horizontal plane intersection. The mean value was 59°. An increase in this value indicated a vertical growth trend, and a reduction, a horizontal growth trend [12].

**Fig. 1:**
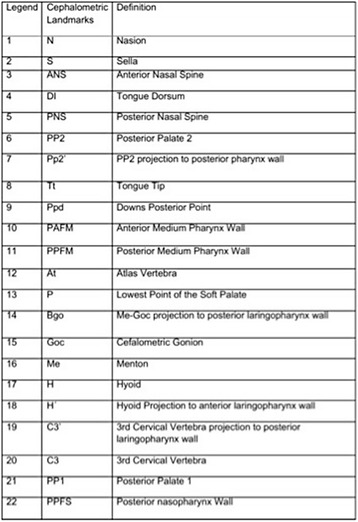
Factors for sleep apnea analysis

**Fig. 2:**
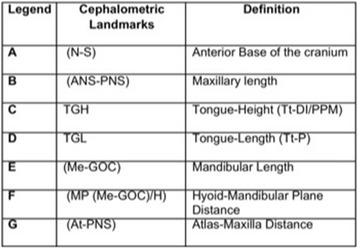
Legend, cephalometric landmarks, and definition for sleep apnea analysis

**Fig. 3:**
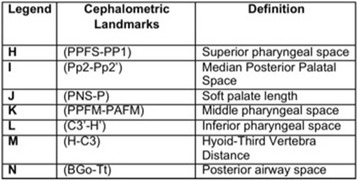
Legend, cephalometric landmarks, and definition

**Fig. 4:**
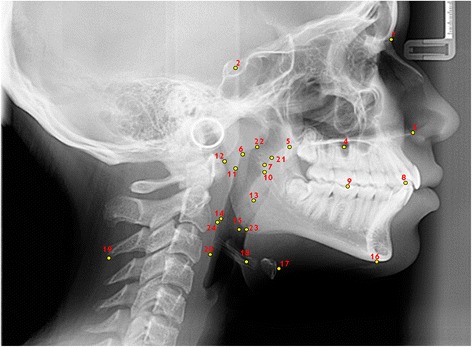
Cephalometric landmarks for sleep apnea analysis

**Fig. 5:**
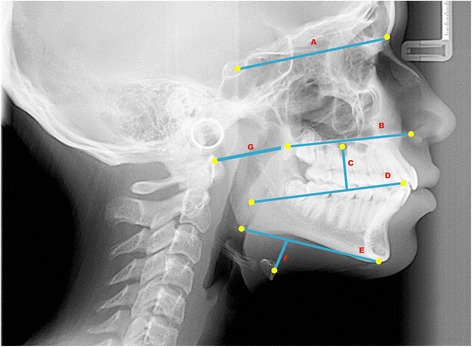
Cephalometric planes (cranial base, maxilla, mandibulla, nasopharynx and hyoid-mandibular plane distance)

**Fig. 6:**
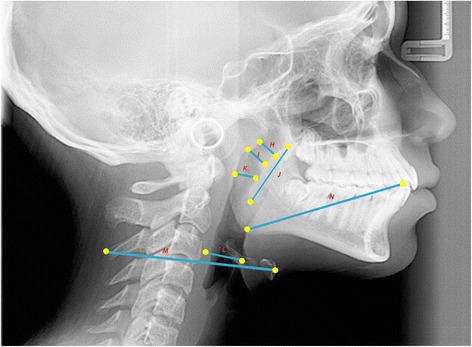
Cephalometric planes (oropharynx and laryngopharynx) for sleep apnea analysis

Fifteen teleradiographs were selected of each craniofacial growth pattern, classified into mesofacial (5 males and 10 females, aged 16–31 years, mean age = 22.21), brachyfacial (8 males and 7 females, aged 17–34 years, mean age = 25.6), and dolichofacial patterns (11 male e 4 females, aged 16–31, mean age = 25.0). To evaluate the upper airways, sleep apnea cephalometry, validated for Brazilians [8], was used, containing 28 points that compose 14 factors (Figs. 1, 2, 3, 4, 5 and 6).

### Statistical analysis

The groups were compared for each of the 14 factors. For the inferior airway space measurement that presented non normal distribution, the Kruskal-Wallis test was used for inter-group comparison. For the remaining data, whose distribution was normal, one-way ANOVA followed by complementary Tukey tests was used. The significance level was 5%.

## Results

The Intraclass Correlation Coefficient (ICC) showed excellent replicability (0.9636).

In the comparison of the three groups for each of the 14 sleep apnea cephalometric measures, statistically significant difference was verified among the groups for the median posterior palatal space (*p* = 0.020), with the complementary Tukey test pointing out difference between the brachyfacial and dolichofacial groups.

When the measurements presented in Table 1 were analyzed, it was verified that the median posterior palatal measurement in the oropharyngeal region was lower for individuals with a dolichofacial (10.64 ± 1.83) pattern when compared with mesofacial (12.64 ± 2.30) and brachyfacial (12.91 ± 2.74) patterns.

**Table 1:** Mean and standard deviation of the 14 factors of sleep apnea cephalometry and the respective *p* values of the comparison among the groups

Measurements	Brachyfacial		Mesiofacial		Dolichofacial		*p* values
	Mean	SD	Mean	SD	Mean	SD	
Cranial base							
Anterior base of the cranium (N-S)	69.82	5.49	67.86	4.27	66.45	5.34	0.203
Maxilla/mandibulla							
Maxillary length (ANS-PNS)	53.93	3.88	54.12	3.39	53.44	3.87	0.877
Mandibular length (Me-Goc)	70.74	6.27	72.01	6.50	70.96	3.38	0.803
Nasopharynx							
Atlas-maxilla distance (At-PNS)	38.59	4.08	37.74	4.91	35.22	3.25	0.080
Superior pharyngeal space (PPFS-PP1)	15.8	2.27	13.68	2.53	14.09	6.42	0.352
Oropharynx							
Middle pharyngeal space (PPFM-PAFM)	11.42	2.97	13.68	2.53	12.03	4.03	0.474
TGL (tongue length) (Tt-P)	71.51	4.59	69.41	6.58	67.95	5.43	0.186
TGH (tongue height) (Tt-DI/P)	23.85	5.27	22.3	3.45	20.70	3.43	0.129
Median posterior palatal space (Pp2-Pp2’)	12.91^a^	2.74	12.64^ab^	2.30	10.64^b^	1.83	0.020*
Soft palate length (PNS-P)	34.39	8.42	31.07	4.16	33.12	3.96	0.307
Laryngopharynx							
Inferior pharyngeal space (C3’-H’)	16.83	5.33	14.00	4.79	12.79	4.72	0.063
Posterior airway space (BGo-Tt)	14.11	2.69	13.36	4.11	12.31	2.86	0.328
Hyoid-third vertebra distance (H-C3)	76.29	7.62	79.24	6.62	76.57	6.39	0.442
Hyoid-mandibular plane distance (MP (Me-Goc)/H)	18.95	4.37	15.97	4.97	18.74	5.53	0.201

Values marked by distinct letters are significantly different from each other Tukey test (*p* < 0.05)

For the other measurements, there was no statistically significant difference (*p* > 0.05). However, for the dolichofacial group, it was observed that on an average, numerically, the inferior pharyngeal space measurements (12.79 ± 4.72), atlas-maxilla distance (35.22 ± 3.25), and posterior airway space (12.31 ± 2.86) were shown to be smaller in comparison with those of the other facial types.

## Discussion

The contribution of this study was to present the use of a cephalometric analysis, commonly used in orthodontics, with the aim of identifying anatomical changes in the upper airways, which may predispose to respiratory disorders.

Cephalometric performed by means of lateral teleradiography has been shown to be an important instrument in the multidisciplinary field for evaluating the upper airways [13, 14] because it is easily accessible and low cost, highly reproducible, and the individual is submitted to a low dose of radiation [4, 15]. Thus, innumerable studies have sought associations of the physical characteristics related to these airway spaces, as a way of predicting pathologies [13–20].

However, there are studies that use computed tomography for morphological evaluation of the airway spaces, particularly due to the possibility of measuring areas and volumes, which is impossible to do by means of other radiographic exams [21–23]. Therefore, the authors point out that one of the limitations of the present study refer to not measuring the airway volumes, due to the type of exam used for evaluation [24]. There was also difficulty in the methodologies with two-dimensional radiographs when performing superimposition of tracings [25]; however, in the present study, no superimpositions were made. Many studies have evaluated the airways by using lateral cephalograms and associated their dimensions with the vertical skeletal pattern of the face and facial morphology [1, 26, 27]. A recent longitudinal study also used lateral cephalometric radiographs for associating changes in the morphology of the nasopharyngeal space in different facial patterns [28], which did not make this method of evaluation unfeasible.

In this study, the authors opted to use the sleep apnea cephalometry instrument because it has been validated for Brazilians and presents standard values that may be used as reference [8].

The authors were able to identify reduction in the median posterior palatal space in individuals with a dolichofacial pattern. A previous study also observed changes in the dimensions of the upper airway related to the reduction in the medial posterior palatal space in individuals with the obstructive sleep apnea syndrome (OSAS) [8]. This measurement expresses the distance from the soft palate to the posterior wall of the pharynx and has a close relationship with the dimensions of the soft palate. The increased length of which was related to presence of OSAS in other researches [8, 29, 30] and the present study. The highest alteration values in upper airway dimensions in OSAS patients occur in the oropharynx [8] and were related to the reduction of the median posterior palatal space [31]. In individuals with a vertical pattern, the mandible is normally retracted and rotated downwards and backwards, thus diminishing the oropharyngeal space [26] Furthermore, the base of the tongue accompanies the direction of mandibular rotation, being positioned downward and backward, thus the soft palate is in a more retrusive position, diminishing the median posterior palatal space.

Some authors have pointed out that when the nasopharyngeal space was reduced, there would be a tendency towards neuromuscular adaptation, leading to vertical growth of the face that is associated with a dolichofacial pattern [14, 21]. However, in this study, no difference was found in the upper airway dimensions in the nasopharyngeal region in the studied volunteers with this facial type. This could be attributed to different sample characteristics in others studies in which the sample was composed of the youngest participants [1, 26]. This aged group could be more susceptible to narrower nasopharyngeal airway spaces due to adenotonsillar hypertrophy, for example [32, 33]. In addition, authors [1] compared Angle Classes I and II, differently from the present study, in which the sample was composed of only Class I patients.

Obstruction of the upper airways forces the patient to breathe through the mouth, and in addition to OSAS, these factors cause oral dysfunction, such as lip incompetence, low position of the tongue in the floor of the mouth, tongue thrust, and may lead to unbalanced muscle and function [32] disturbances in swallowing, mastication, speech [34], and stability of occlusion [35].

This study was performed using two-dimensional digital lateral cephalogram that is a limitation. Therefore, it is important to recognize that three-dimensional evaluation of the airways by means of cone-beam computed tomography, respecting legal and ethical aspects, due a higher dose of radiation, could be useful for improved assessment in further studies to minimize this limitation.

## Conclusions

Under the conditions of this study and considering the results, the authors could conclude that there were no differences in nasopharyngeal and hypopharyngeal airway spaces in brachyfacial and mesofacial individuals. Oropharyngeal space reduction was observed in Angle Class I dolichofacial individuals, characterized by reduction in the median posterior palatal measurement.
